# Gestational trophoblastic neoplasia with extrauterine metastasis but lacked uterine primary lesions: a single center experience and literature review

**DOI:** 10.1186/s12885-022-09620-2

**Published:** 2022-05-06

**Authors:** Jingnan Li, Yu Wang, Bingjian Lu, Weiguo Lu, Xing Xie, Yuanming Shen

**Affiliations:** 1 School of Medicine, Women’s Hospital, Zhejiang University, Hangzhou, 310058 China; 2 Department of Gynecologic Oncology, School of Medicine, Women’s Hospital, Zhejiang University, Hangzhou, 310006 China; 3 Center of Uterine Cancer Diagnosis & Therapy of Zhejiang Province, Hangzhou, 310006 China; 4 Cancer Center, Zhejiang University, Hangzhou, 310058 China

**Keywords:** Gestational trophoblastic neoplasia, Neoplasm metastasis, Choriocarcinoma, Diagnosis, Therapy

## Abstract

**Background:**

To investigate the clinicopathological characteristics, diagnoses, treatments, and outcomes of a special type of gestational trophoblastic neoplasia (GTN) which only has extrauterine metastases without uterine primary lesions.

**Methods:**

The medical records and pathological sections of the patients who were pathologically diagnosed as GTN, only had extrauterine metastatic lesions but lacked uterine primary lesions, in Women’s Hospital of Zhejiang University School of Medicine from February 2014 to March 2021 were collected and reviewed.

**Results:**

Thirteen patients with pathologically confirmed GTN presenting with extrauterine metastases from a missing primary site were included in the past 7 years. The median age was 31.2 years old. 76.9% of patients had a non-hydatidiform pregnancy last time. The intervals between the antecedent pregnancy were > 12 months in 61.5% of patients. Pretreatment serum human chorionic gonadotropin(hCG) levels ranged from 118.7 to 807,270 IU/L. Six patients were misdiagnosed as ectopic pregnancy at initial diagnosis, and 4 as primary tumors at metastatic sites. All of them were diagnosed definitely by surgical pathology including 8 choriocarcinomas (CC), 4 epithelioid trophoblastic tumors (ETTs), and 1 mixed GTN (CC mixed with ETT). All patients achieved complete remission (CR) after treatments. Three patients relapsed; no patient died by the end of follow-up.

**Conclusion:**

GTN presenting with extrauterine metastases from a missing primary site is easily misdiagnosed. Detection of serum hCG in these patients can reduce misdiagnosis. Chemotherapy combined with individualized surgery should be considered for these special GTN patients. Immune checkpoint inhibitors might be potential remedial measures for refractory and recurrent patients.

**Supplementary Information:**

The online version contains supplementary material available at 10.1186/s12885-022-09620-2.

## Introduction

Gestational trophoblastic neoplasia (GTN) is a group of tumors derived from abnormal proliferative placental trophoblastic cells. According to the 2010 World Health Organization (WHO) classification [1], GTN is classified histologically into a series of pregnancy-related malignancies, including, gestational choriocarcinomas (CC) and placental site trophoblastic tumor (PSTT), and epithelioid trophoblastic tumor (ETT) [2, 3].

GTN can be divided into non-metastatic GTN and metastatic GTN. Non-metastatic GTN refers to lesions confined to the uterus. Metastatic GTN refers to lesions occurring outside the uterus, typically hematogenous spreading. The lung is the most common site of metastasis, and other metastatic sites include the vagina, tubes, ovaries, liver, spleen, kidneys, bowel, brain, etc. [4] Distant metastases such as liver and brain are more likely to have a poor prognosis [5]. The majority of metastatic GTN presented as a primary uterine lesion complicated with extrauterine metastases. However, a few metastatic GTN only presented with extrauterine metastases and the primary lesion was missed. Such metastatic GTN is rare in clinical practice, and most cases are reported as a single case. The first symptoms are usually different due to different metastatic sites and lack of clinical manifestations related to GTN, making diagnosis difficult and easy to misdiagnose.

Thus, it is necessary to conduct retrospective studies aimed at exploring the clinicopathological data, diagnosis, treatment, and prognosis in patients of this kind of GTN, to provide a reference for diagnosis and treatment of this rare entity.

## Materials and methods

### Patients and data collection

All patients, who were pathologically diagnosed as GTN presenting with extrauterine metastases from a missing primary site in Women’s Hospital of Zhejiang University School of Medicine from February 2014 to March 2021, were collected using computerized databases from the Departments of Gynecologic Oncology and Pathology. Inclusion criteria: 1) New diagnosed GTN patients; 2) Extrauterine metastases were diagnosed with GTN by histopathology and immunohistochemistry; 3) No primary uterine lesions were found in the evaluation of gynecological examination combined with transvaginal ultrasound of uterine adnexa, abdominal and pelvic Computed Tomography (CT), with or without pelvic Magnetic Resonance Imaging (MRI). Exclusion criteria: The patients had a history of other malignant tumors. The medical records were reviewed and the following data were collected, including age, symptoms, previous pregnancy, pre- and post-treatment serum human chorionic gonadotropin (hCG) levels, metastasis site, primary clinical diagnosis, pathological diagnosis, stage and prognosis score, treatments, recurrence, and prognosis.

Federation International of Gynecology and Obstetrics (FIGO) 2000 stage was used for staging, and World Health Organization (WHO) prognostic index score standard (2014) was used for prognostic score [3].

The study was approved by the Ethics Committee of the Women's Hospital of Zhejiang University School of Medicine. This study was a retrospective analysis of clinical data, unrelated to human bioethics. Informed consents were obtained for all follow-up contents. the study was performed under the principles of the 2013 Declaration of Helsinki [6]. All methods were performed under the relevant guidelines and regulations.

### Immunohistochemistry

Programmed death ligand 1(PD-L1) expression was detected by Immunohistochemistry in all these patients. Mouse anti-human PD-L1 antibody (ab210931; Abcam, Shanghai, China) diluted at 1:100 was used for detection. Following protocols, the two-step EnVision immunostaining procedure (Dako, Carpentaria, CA) was performed. The normal human placenta was regarded as the positive control, and PD-L1 negative cervical squamous cell carcinoma was the negative control. Membrane staining was considered positive. The results were confirmed by pathologists and evaluated by tumor proportion score (TPS) and combined positive score (CPS) with reference to our previous study [7].

### Follow-up and outcome

All patients were followed via telephone interviews or at clinics. Complete Remission (CR) was defined as a continuous normalization of serum hCG levels for at least 4 weeks after chemotherapy, while resistance was defined as an increase of hCG levels > 10% or stabilization of ± 10% within 2 weeks after two courses of chemotherapy. Relapse was defined as hCG levels rising again after three months of CR in the absence of a normal pregnancy. Mixed GTN was defined as the coexistence of choriocarcinoma and/or PSTT and/or ETT components. Undetermined GTN was defined as group serum β-hCG levels elevated in the absence of the pregnancy less than 3 months after completed treatment [8-11].

### Statistical analysis

Statistical analysis was performed using SPSS26.0 for Windows (IBM Corporation, Armonk, NY, USA). Continuous data were described as mean ± SD (standard deviation) or median, and categorical data as frequency and percentage. Fisher's exact test was used to check qualitative variables, and *P* < 0.05 was considered statistically significant.

## Results

### Clinical characteristics

A total of 13 patients with pathologically confirmed GTN presenting with extrauterine metastases from a missing primary site were included, and their clinicopathological characteristics, diagnosis, treatments, and outcomes were summarized in Table 1. The mean age at diagnosis was 30.5 ± 5.8 years (19–41 years). Imageology ± laparoscopy confirmed that all patients had no uterine lesions throughout the course of the disease. Antecedent pregnancies were molar pregnancy in 3 patients (23.1%), abortion in 8 patients (61.5%), term pregnancy in 1 (7.7%) patient, coexisting pregnancy in 1 (7.7%) patient. In 70% of molar and abortion cases, the nature of the pregnancy substance was confirmed. After an abortion, the serum hCG level was followed up once a week until the value was normal twice in a row. Most cases were reviewed regularly after abortion, except Cases 7, 8, and 11.

**Table 1:** The clinical characteristics, treatment and outcomes of 13 patients with metastatic GTN without primary lesions

Patients No	Age	G/P	Symptoms	Signs	Antecedent pregnancy	Interval (months)	Pretreatment hCG(IU/L)	Initial disposition	Metastasis site
1	28	3/1	Vaginal bleeding	Lung mass	Mole	17	2021	GTN	Right lung
2	31	1/0	Vaginal bleeding	Lung mass	Mole	3	121	GTN	Right lung
3	23	2/0	None	Lung mass	Abortion	11	277	Ectopic pregnancy	Right lung
4	32	4/2	Vaginal bleeding, cough	None	Term	3	194	Lung cancer	Right lung
5	30	1/0	Vaginal bleeding	Lung mass	Mole	14	144	GTN	Right lung
6	27	2/0	Vaginal bleeding	Lung mass	Abortion	36	967	Ectopic pregnancy	Right lung
7	36	3/1	Vaginal bleeding	None	Abortion	60	1100	Ectopic pregnancy	Left lung
8	31	1/0	Vaginal bleeding	Ovarian mass	Abortion	108	612	Ectopic pregnancy	Left ovary
9	19	0/0	Vaginal bleeding	Ovarian mass	Abortion	N/A	104,400	Ectopic pregnancy	Right ovary
10	37	2/0	None	Lung and breast mass	Abortion	60	21,136	Lung cancer, breast cancer	Left lung, kidney, brain, breast, liver, waist quadratus
11	41	5/2	None	Adnexal mass	Abortion	5	> 2000	Ectopic pregnancy	Right pelvic funnel ligament and mesocolon
12	36	5/1	Abdominal pain	Adnexal mass	Abortion	34	119	Ovary tumor	Masses on the right ovary, omentum, and abdominal wall
13	26	0/0	None	Ovarian and liver masses	Pregnancy (29 weeks)	7	807,270	Ovary tumor	The right lung, pelvic abdomen, liver

Patients No	Number of metastases	Initial treatment	Definitive diagnostic method	Pathological diagnosis	FIGO stage	Prognostic score	Chemotherapy	The number of consolidation chemotherapy	Relapse
1	1	5 cycles of TP	VATS	ETT	III	/	2 cycles of EP-EMA + 5 cycles of EP	5	No
2	1	D&C	VATS	CC	III	5	6 cycles of MTX	N/A	No
3	1	D&C + Laparoscopy	VATS	ETT	III	/	3 cycles of EP-EMA	3	No
4	1	D&C + Laparoscopy	VATS	ETT	III	/	3 cycles of EP-EMA	3	No
5	1	PTNB	VATS	ETT	III	/	3 cycles of EMA-CO	3	No
6	1	PTNB	VATS	CC	III	5	6 cycles of MTX	3	Yes
7	1	D&C + Laparoscopy + 1 cycle of MTX	VATS	CC	III	7	3 cycles of TP	3	No
8	1	Laparoscopy + 1 cycle of MTX	LOCR	CC	II	7	3 cycles of EMA-CO + 3 cycles of EP-EMA + 4 cycles of FAEV	5	Yes
9	1	Laparoscopy	LOCR	CC	II	> 7	7 cycles of EMA-CO	3	No
10	Multiple	VATS + SM	VATS + SM	CC	IV	17	2 cycles of EMA-CO + 8 cycles of EP-EMA + 2 cycles of MTX intrathecal injection	5	Yes
11	Multiple	Laparoscopy + 2 cycles of MTX	LMR	CC	II	9	3 cycles of EMA-CO + 2 cycles of EMA	3	No
12	Multiple	D&C + Laparoscopy	LUA + LMR	CC/ETT	IV	12	4 cycles of EP-EMA	3	No
13	Multiple	Cesarean	Cytoreductive surgery	CC	IV	7	5 cycles of EMA-CO	N/A	No

*G/P*, Gravida/para, *hCG*, Human chorionic gonadotropin; *IU/L*, International units per liter; *FIGO*, International Federation of Obstetrics and Gynecology; *GTN*, Gestational trophoblastic neoplasm; *D&C*, Dilation and curettage; *PTNB*, Percutaneous needle biopsy of the chest;* VATS*, Video-assisted thoracoscopic surgery; *SM*, Segmental mastectomy; *LOCR*, Laparoscopic ovarian cyst resection; *LUA*, Laparoscopic unilateral adnexectomy; *LMR*, Laparoscopic mass resection; *ETT*, Epithelioid trophoblastic tumor; *CC*, Choriocarcinoma; *TP*, Paclitaxel, Cisplatin; *MTX*, Methotrexate;* EP-EMA*, Etoposide, Methotrexate, Actinomycin-D/Etoposide, Cisplatin; *EP*, Etoposide, Cisplatin; *EMA-CO*, Etoposide, Methotrexate, Actinomycin-D/Cyclophosphamide, Vincristine;* FAEV*, Floxuridine, Actinomycin-D, Etoposide, Vincristine; None, no positive symptoms and signs; *N/A*, not available. (The patients returned to the local hospital for treatment after HCG values returned to normal levels, and the regimen and cycles of consolidation chemotherapy are uncertain. Follow-up showed that hCG was reduced to normal.) /, ETT does not apply to the FIGO scoring system and is not rated

**Fig. 1:**
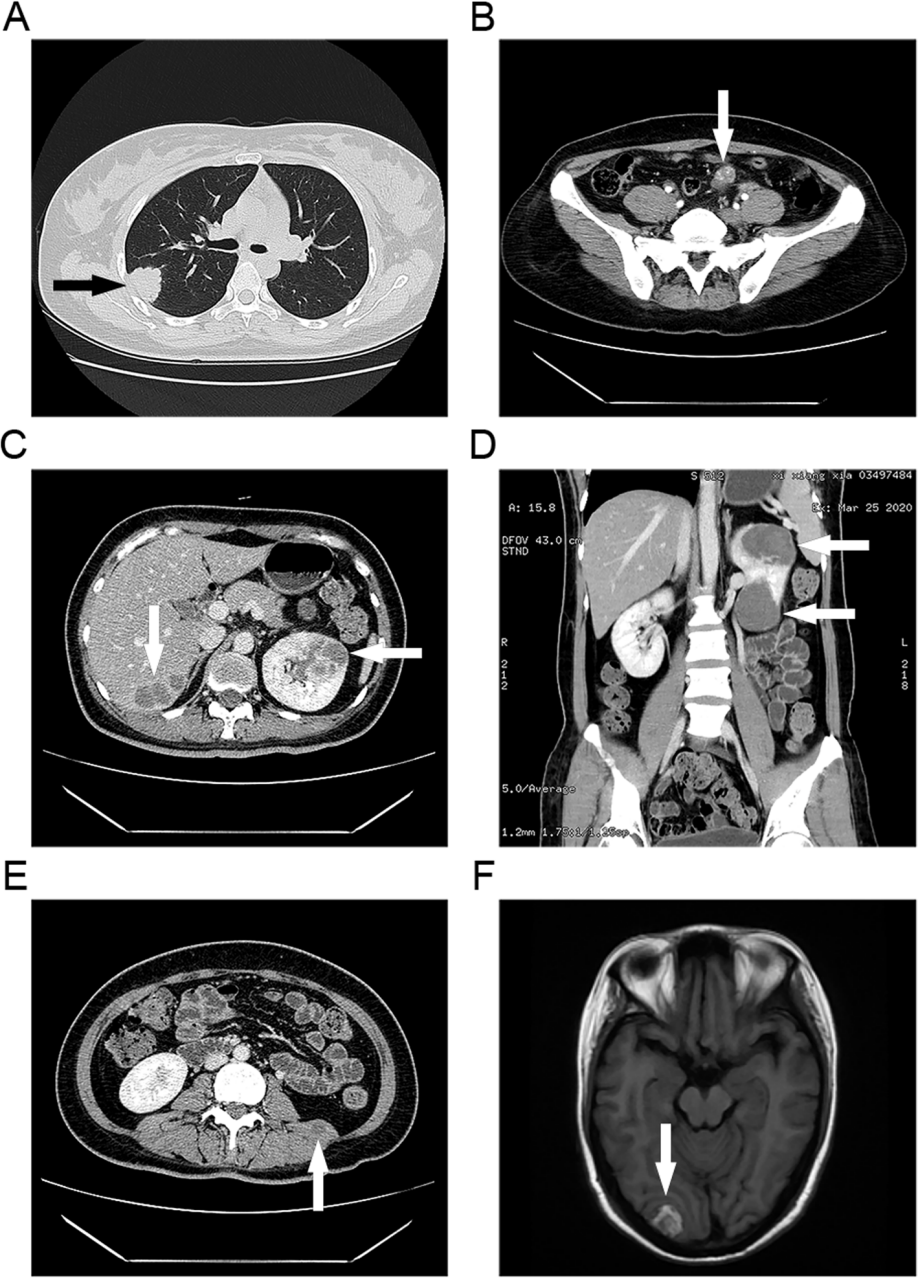
Imaging manifestations of extrauterine lesions in GTN

The interval between the antecedent pregnancy and treatment was ≤ 12 months for 5 patients including 1 patient during pregnancy and > 12 months in the remaining 8 patients. Pretreatment serum hCG levels ranged from 118.7 to 807,270 IU/L. Notably, 10 of 13(76.9%) patients had levels of hCG ≤ 2,500 IU/L. Metastases in one or more organs were the most common symptom which was reported in all of the 13 patients including 7 (53.8%) patients presented with pulmonary nodules, 2 (15.4%) patients presented with an adnexal mass, and another 4(30.8%) patients presented with masses in multiple organs including kidney, liver, brain, pelvic cavity, and ligaments, etc. In addition, all metastases presented as localized hemorrhagic necrosis as shown in Fig. 1. Abnormal vaginal bleeding was the second common symptom which was reported in 8 (61.5%) patients. Other presentations included abdominal pain and amenorrhea.

(A) The chest CT image of case 4 showed a lobulated mass (black arrowhead) about 2.6*3.1 cm in size in the right upper lung. (B) Abdominal contrast-enhanced CT image of case 11 showed an irregular mass (white arrowhead) near the left ovary, about 4.3*2.7*2.6 cm in size, with uneven internal enhancement on the contrast-enhanced scan. (C) (D) (E) Abdominal enhanced CT image of the case 10 suggested multiple lesions (white arrowhead) in the liver, left kidney, and left quadratus lumbago muscle, which were considered as metastatic tumors. (F) The cranial enhanced MRI image in case 10 showed multiple nodules in the brain parenchyma (the largest one is shown here, about 1.8 cm in length), and metastasis was considered.

### Diagnosis and treatments

Based on the final operative histopathology results, the 13 patients have confirmed their diagnosis as follows: 8 CC (61.5%), 4 ETTs (30.8%), and 1 mixed GTN (CC mixed with ETT) (7.7%). However, the initial diagnosis of all the 13 patients was incorrect or unclear. Six patients were misdiagnosed as ectopic pregnancy, 2 as the ovarian tumor,1 as lung cancer,1 as breast and lung cancer, and the remaining 3 suspected as GTN. During the definitive diagnosis process, 9 patients received multiple surgeries which involved dilation and curettage (D&C) and video-assisted thoracoscopic surgery (VATS) in 1 patient (case 2), D&C combined with laparoscopy and VATS in 3 patients (case 3, 4, 7), percutaneous needle aspiration biopsies of lungs (PTNB) and VATS in 2 patients (case 5, 6), VATS and segmental mastectomy (SM) in 1 patient (case 10), laparoscopy and laparoscopic mass resection (LMR) in 1 patient (case 11), D&C and VATS combined with laparoscopic unilateral adnexectomy (LUA) and laparoscopic mass resection (LMR) in 1 patient (case 12). Other 4 patients were confirmed for GTN by single surgery including case 1 patient by VATS, cases 8 and 9 by laparoscopic adnexal lesions resection, and case 13 by cytoreductive during a cesarean. For related operations other than obstetrics and Gynecology, we invited doctors from all relevant departments to assist us in completing them.

After surgical confirmation and removal of the GTN lesion, all 13 patients underwent further chemotherapy. According to WHO 2000 risk score standard, 2 low-risk CC patients initially received Methotrexate (MTX) regimen, 6 high-risk CC patients initially received TP (Taxol and Carboplatin) or EMA-CO (Etoposide, Methotrexate, Actinomycin-D/Cyclophosphamide, and Vincristine) regimen respectively, 1 ultra-high-risk CC patient initially received EP-EMA (Etoposide, Methotrexate, Actinomycin-D/Etoposide, and Cisplatin) regimen, 4 ETT patients have initially received EP-EMA or EMA-CO regimen. Case 2 presented severe oral ulcers with infection. Bone marrow suppression was found in other patients during chemotherapy, and abnormal liver function was also found in patients 4, 5, 8, 9, and 11.

### Recurrence and prognosis

All patients had a CR after treatment. The median duration of follow-up was 24.5 months (5–85.2 months), and 3 (case 6, 8, 10) patients had a recurrence within 2 to 5 months, no patients died. Details as shown in Table 2. The case 6 had no vaginal bleeding, cough, abdominal pain, and other discomforts. The patient experienced CR again after a VATS operation by thoracic surgeons to remove the lung lesion and 3 cycles of EMA-CO as consolidation chemotherapy. The case 8 also had no complaints of discomfort. The patient was reexamined with elevated HCG and left ovarian abnormality. She experienced CR again after laparoscopic left adnexectomy and 5 cycles of TP regimens. In case 10, hCG increased again and epilepsy was found after hCG was negative for more than three months. Cranial MRI enhanced scan showed hemorrhage of brain metastasis. She was transferred to a general hospital for gamma-knife, and postoperative pathology considered CC with hemorrhage and necrosis of brain metastases. Then she was given weekly treatment with Albumin Paclitaxel combined with PD-1 immunotherapy (Tirelizumab) for 5 cycles and achieved CR again, then Tirelizumab maintenance therapy. Well controlled by follow-up.

**Table 2:** The management and outcome of the relapse patients

Patients No	Relapse	Symptoms	Signs	Pretreatment hCG(IU/L)	Interval times after hCG negative (months)	Surgery	Pathological diagnosis	Treatment
6	1	None	Persistent right lung mass	20.6	3	VATS	CC with pulmonary metastasis	3 cycles of EMA-CO
8	1	None	Left ovary abnormality	180.9	5 +	LUSO	CC	5 cycles of TP
10	1	Epilepsy, convulsions	Brain mass	10.9	3 +	Gamma knife	CC with brain metastasis	Weekly Albumin Paclitaxel combined with PD-1 immunotherapy (Tirelizumab) for 5 cycles, Tirelizumab maintenance therapy

*HCG*, Human chorionic gonadotropin, *IU/L*, International units per liter; *VATS*, Video-assisted thoracoscopic surgery; *LUSO*, Laparoscopic unilateral salpingo-oophorectomy; *CC*, Choriocarcinoma; *EMA-CO*, Etoposide, Methotrexate, Actinomycin-D/Cyclophosphamide, Vincristine; TP, Paclitaxel, Cisplatin

Only one patient (case 9) had reproductive requirements who had delivered 2 healthy boys in 2017 and 2019 respectively. No recurrence of the GTN was observed.

Univariate analysis showed that age, antecedent pregnancy, interval months from index pregnancy, pretreatment serum hCG level, site and number of metastases, prognosis score, and pathological type were not associated with recurrence (supplyment1).

### PD-L1 expression

**Fig. 2:**
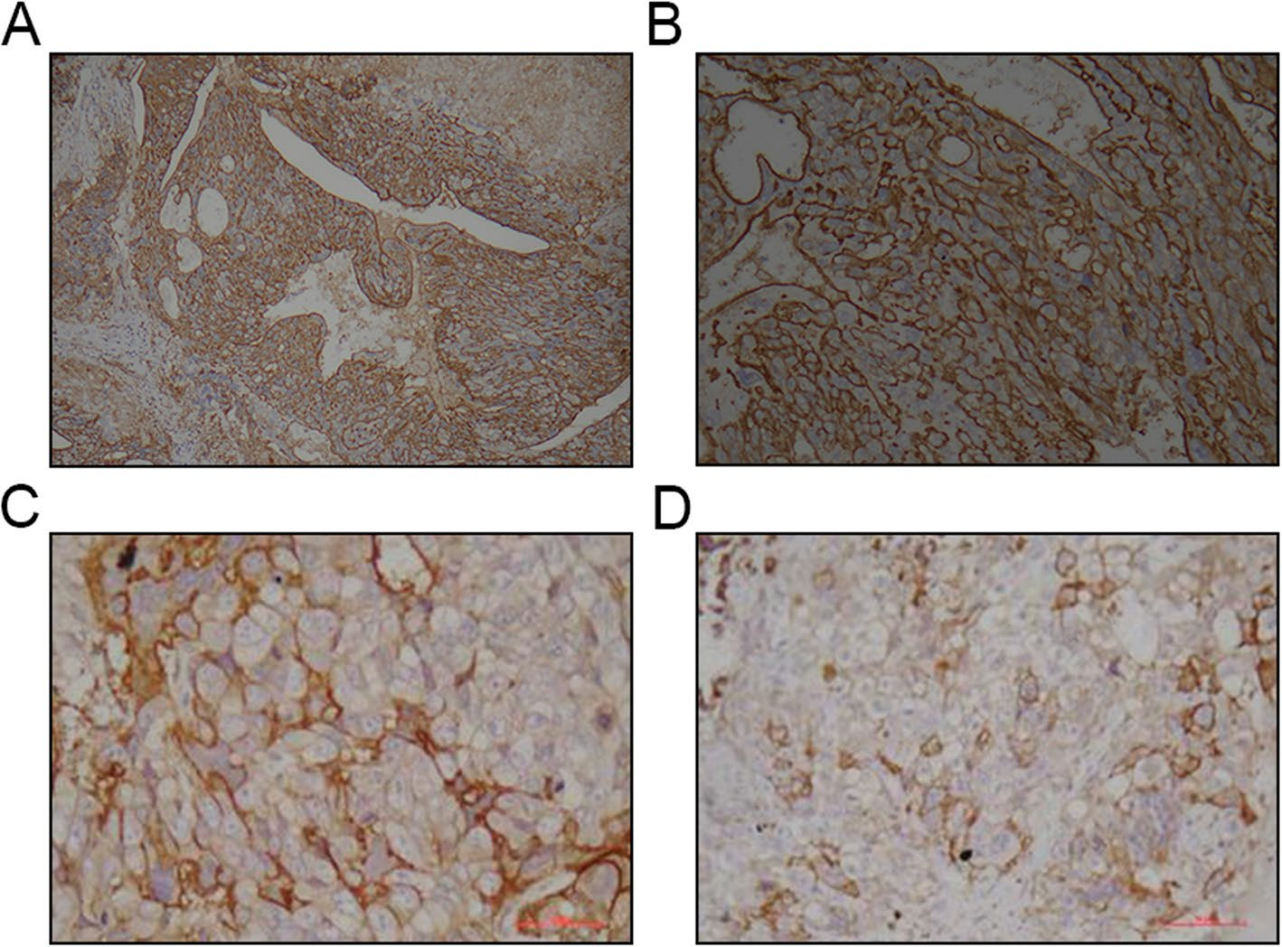
Immunohistochemistry images

All available GTN tissue samples from patients were tested for PD-L1 expression, showing strong positive staining. Positive immunohistochemistry staining images were shown in Fig. 2.

(A) (B) Pelvic mass tissue, choriocarcinoma, Immunohistochemistry showed diffuse membrane staining, PD-L1 expression > 50% (magnification, 100 × and 200x). (C) Lung tissue, PD-L1 positive, TPS > 50%, CPS > 50% (magnification, 400x). (D) Breast tissue, PD-L1 positive, TPS > 50%, CPS > 50% (magnification, 400x).

## Discussion

The 13 cases accounted for 0.04% of the total cases of gynecological oncology in our hospital, and 6.5% of the total cases of gestational trophoblastic tumors from February 2014 to March 2021. Metastatic GTN without primary lesion is rare in clinical practice, and mostly described in case reports, little is known about its clinical characteristics, which is easy to be misdiagnosed and mistreated. At present, the occurrence of this special GTN has been speculated as the primary uterine lesion is too small or grows slowly after tumor metastasis, which cannot be detected by existing imaging techniques, or the primary lesion is cleared by the autoimmune system after metastasis. For this purpose, we performed a retrospective study and literature review to better understand these special GTNs.

Aside from the 13 cases in our retrospective study, other 12 cases of extrauterine metastatic GTN without primary lesion were obtained by the literature reviewed [12–20]. The characteristics, initial diagnoses, treatments, and outcomes of the 12 patients in the literature were summarized in Table 3. The mean age at diagnosis was 39.4 years (25–56 years). Antecedent pregnancies were completed to term in 3 (25%) patients, ended in mole pregnancy in 3(25%) patients, ended in abortion in 1 (8.3%) patient, and unknown in other 5 (41.7%) patients. The interval between the antecedent pregnancy and treatment was from 2 weeks to 16 years. Pretreatment serum hCG levels ranged from 6.9 to 610,000 IU/L. However, only 2 of 12 patients had high levels of hCG > 2,500 IU/L. Hemorrhagic necrotic metastases in one or more organs were the most common symptoms, usually in the lung (83.3%). Other symptoms were atypical and varied with different sites of metastasis, such as vaginal bleeding, abdominal pain, hemoptysis, etc. The vast majority of patients were misdiagnosed at the initial diagnosis,1 patient was misdiagnosed as ectopic pregnancy and 8 patients were misdiagnosed as a primary tumor at the metastatic site. Most patients were diagnosed surgically, 5 patients were diagnosed by thoracoscopy, 1 by vaginal mass resection, 1 by laminectomy, 1 by external iliac lymph node biopsy, and 1 by total hysterectomy. Among the 12 patients, 8 patients were histopathological confirmed ETT, 4 were CC. Among the 8 patients whose data were collected, 6 were alive without disease,1 relapsed after remission and yet was alive after salvage treatment, one died. Most patients received EMA-CO or EP-EMA regimen; 1 patient received an immune checkpoint inhibitor (pembrolizumab) for treatment.

**Table 3:** The clinical data of patients with metastatic GTN without primary lesions reported in the literature

Author	Age	G/P	Symptoms	Signs	Antecedent pregnancy	Interval times	Pretreatment hCG(IU/L)	Initial diagnosis	Metastasis site	Number of metastases	Diagnostic method	Pathological diagnosis	FIGO stage and Prognostic score	Subsequent treatment	Outcomes
Lei et al	49	1/1	None	Lung mass	Mole	N/A	N/A	N/A	Lung	1	VATS	ETT	/	N/A	NED
Ru et al	33	3/1	None	Vaginal mass	Abortion	6 months	70	N/A	Vaginal wall, lung	N/A	Mass excision	ETT	/	2 cycles of EMA-CO	NED
Chohan et al	36	4/4	Severe low back pain	T10 radiculopathy	Term	2 weeks	16	Infiltrating tumor	Spine, liver, lung	Multiple	Laminectomy	ETT	/	EMA-CO	DOD
Urabe et al	38	5/4	Poor physical condition	Lung mass	Term	N/A	80.1	CC	Lung	Multiple	VATS	ETT	/	6 cycles of EMA-CO, thoracoscopy	Relapse
Hamazaki et al	32	3/1	None	Lung mass	Mole	5 years	N/A	Lung cancer	Lung	1	VATS	ETT	/	N/A	NED
Hamazaki et al	42	2/2	Hemoptysis and cough	Small nodules in lung and liver	N/A	N/A	N/A	Lung cancer	Lung, liver	Multiple	VATS	ETT	/	Chemotherapy	NED
Bell et al	47	2/2	Recurrent respiratory infections	Pulmonary emboli, pulmonary nodules, and infarcts	N/A	12 years	9	N/A	Lung, the infrahilar mass, rectus abdominis muscles, a right external iliac lymph node	Multiple	External iliac lymph node biopsy	ETT	/	7 cycles of EP-EMA, 29 times of pembrolizumab	NED
Davis et al	49	N/A	None	None	Term	16 years	6.9	N/A	Intra-abdominal	Multiple	TAH, BSO, tumor debulking	ETT	/	EP-EMA, prior to surgery	NED
Whaley et al	56	N/A	Metastatic cervical carcinoma with liver hemorrhage	None	N/A	N/A	5365.1	N/A	Liver	N/A	N/A	CC	IV	N/A	N/A
Whaley et al	40	N/A	Splenic injury with liver hemorrhage	None	N/A	N/A	13.4	N/A	Liver	N/A	N/A	CC	IV	N/A	N/A
Whaley et al	25	N/A	Retroperitoneal bleed	None	N/A	N/A	610,000	N/A	Lung	N/A	N/A	CC	III	N/A	N/A
Maruoka et al	26	N/A	High level of hcg, a pulmonary nodule	Lung mass	Mole	6 years	233.8	Ectopic pregnancy	Lung	1	VATS	CC	III; 6	N/A	N/A

*N/A*, Not available; None, no positive symptoms and signs; G/P, Gravida/para; *hCG*, Human chorionic gonadotropin; *IU/L*, International units per liter; *FIGO*, International Federation of Obstetrics and Gynecology; *CC*, choriocarcinoma; *ETT,* epithelioid trophoblastic tumor; VATS, video-assisted thoracoscopic surgery; TAH, total abdominal hysterectomy; BSO, bilateral salpingo-oophorectomy; /, ETT does not apply to the FIGO scoring system and is not rated. EMA-CO, Etoposide, Methotrexate, Actinomycin-D/Cyclophosphamide, Vincristine;* EP-EMA*, Etoposide, Methotrexate, Actinomycin-D/Etoposide, Cisplatin. *NED*, no evidence of disease; *DOD*, dead of disease

Based on our cohort and cases reported in the literature, it showed that this special GTN was a rare particular type of GTN which different from ordinary GTN in clinical presentation, histopathology, diagnosis, and treatment. This particular type of GTN had the following clinical presention:1) Most antecedent pregnancies were non-hydatidiform mole; 2) It always presented as hemorrhagic necrotic metastases in one or more organs rather than abnormal vaginal bleeding; 3) Serum hCG level can be slightly elevated in the majority patients and exceed 2, 500 IU/L in a very few patients. Due to the lack of typical clinical presentations, this particular type of GTN was easily misdiagnosed as the primary tumor at the metastatic site at the first diagnosis, and surgical diagnosis is often required. Thus, for women with the above manifestations, serum hCG should be tested to avoid misdiagnosis.

ETT and CC were the main pathological types in this special GTN which were confirmed by surgical histopathology. Patients with single lung metastases and low hCG levels were more likely to have ETT, accounting for 20% of the 25 patients. As we know, the ordinary GTN is a solid tumor diagnosed and treated based on clinical evidence without a histological diagnosis. The treatment principle is chemotherapy, supplemented by surgery [21]. Chemotherapy regimens depend on stage and score, with low-risk patients (GTN < 7) receiving single-agent chemotherapy first, high-risk patients (GTN ≥ 7) receiving combination regimens, and surgery being used only for removal of uncontrolled bleeding or drug-resistant lesions. However, surgery was of great value in this particular type of GTN patient, which could not only confirm the diagnosis but also play an important role in the treatment. In this group, 10 patients were cured by surgery, of which 30% were drug-resistant lesions removed and 70% were ETT whose hCG was reduced to normal range after confirmed surgery. EMA-CO and EP-EMA were the first choices for chemotherapy. 41.7% (5/12) of the cases with available data in the literature received remission after treatment with EMA-CO or EP-EMA, and 3 of the 13 (23.1%) patients in this group were resistant to both MTX single-agent chemotherapy and TP for the first time, but still achieved complete remission after treatment with EMA-CO or EP-EMA. Thus, we suggested that obtaining pathology was an important way to guide the treatment, and EMA-CO or EP-EMA chemotherapy combined with individualized surgical treatment was the first choice for this special GTN.

Of the 12 patients reported in the literature, 1 patient recurred and 1 patient died; of the 13 patients in our cohort, 3 patients relapsed and none died. As the literature reported that the recurrence rate of conventional low-risk and high-risk GTN was 1.6–3.1% and 6.9% respectively [22-24]. In our study, the recurrence rate of this special GTN was 16% which was slightly higher than that of ordinary GTN, which may be related to the prolonged treatment time, the metastasis of important organs, and the insensitive to chemotherapy for ETT. Fortunately, the overall prognosis of these patients was generally good, most patients with recurrence could still achieve CR after surgery and chemotherapy.

Two patients (case 10 and Bell etc. [17]) were finally treated with PD-1 antibody which could control the disease well. The refractory and recurrent cases achieved CR with chemotherapy combined with immune checkpoint inhibitors (pembrolizumab) based on the high expression of PD-1/PD-L1 [6, 25]. Studies showed that about 30%-40% of high-risk patients responded poorly to first-line therapy or relapsed after remission. Remedial chemotherapy with Etoposide and Platinum drugs, which can be combined with surgical excision of drug-resistant lesions, has a good cure rate. The chemotherapy regimen included TP/TE (Paclitaxel and Cisplatin interchanged weekly with Paclitaxel and Etoposide), BEP (Bleomycin, Etoposide, Cisplatin), VIP (Etoposide, Ifosfamide, Cisplatin), and ICE (Ifosfamide, Carboplatin, Etoposide) [9, 26]. The potential role of immunotherapy deserves further investigation. The role of high-dose chemotherapy (HDC) and peripheral blood stem cell support is uncertain [27]. The significant advances in immunotherapy in recent years, alongside the fact that GTN strongly expresses PD-L1 has led to checkpoint inhibitor use in GTN [28]. Pembrolizumab (anti-PD-1) has effectively induced complete responses in 75–80% of unresectable, chemo-resistant GTN including cases that had failed high dose chemotherapy. Here, we firstly proved that immune checkpoint inhibitors could be potential salvage measures for this special GTN.

We have to acknowledge the limitation of this study that all patients did not undergo hysterectomy so we can’t histopathologically confirm no lesion at the primary uterine site. Meanwhile, we need to consider the possibility that patients are non-gestational trophoblastic tumors rather than gestational trophoblastic tumors.

Although this study has a retrospective limitation and relatively small sample size, our findings represent the largest cohort of patients with only extrauterine metastases without primary lesions and provide unique information for the clinical treatment of these patients.

## Conclusion

In conclusion, improving the understanding of this special GTN, avoiding misdiagnosis and mistreatment, chemotherapy combined with individualized surgical treatment was the key to improving the prognosis of patients. Immune checkpoint inhibitors might be potential remedial measures for refractory and recurrent patients.

## Supplementary Information

**Additional file 1. Supplement 1: **The univariate correlation analysis of metastatic GTN without primary tumor and the outcome of recurrence. 

## Data Availability

All data generated or analyzed during this study are included in this published article (and supplementary information files).

## Abbreviations

GTN: Gestational trophoblastic neoplasia.
WHO: World Health Organization
IHM: Invasive hydatidiform mole
CC: Choriocarcinomas
PSTT: Placental site trophoblastic tumor
ETT: Epithelioid trophoblastic tumor
CT: Computed Tomography
MRI: Magnetic Resonance Imaging
hCG: Serum human chorionic gonadotropin
FIGO: Federation International of Gynecology and Obstetrics
PD-L1: Programmed death-ligand 1
TPS: Tumor proportion score
CPS: Combined positive score
CR: Complete remission
D&C: Dilation and curettage
VATS: Video-assisted thoracoscopic surgery
PTNBL: Percutaneous needle aspiration biopsies of lungs
SM: Segmental mastectomy
LMR: Laparoscopic mass resection
LUA: Laparoscopic unilateral adnexectomy
MTX: Methotrexate
TP: Taxol, Carboplatin
EMA: CO Etoposide, Methotrexate, Actinomycin-D/Cyclophosphamide, Vincristine
EP-EMA: Etoposide, Methotrexate, Actinomycin-D/Etoposide, Cisplatin
TP/TE: Paclitaxel and Cisplatin interchanged weekly with Paclitaxel and Etoposide
BEP: Bleomycin, Etoposide, Cisplatin
VIP: Etoposide, Ifosfamide, Cisplatin
ICE: Ifosfamide, Carboplatin, Etoposide
HDC: High dose chemotherapy
